# Letter to the Editor: Ketamine for pain management: let's not neglect practical concerns

**DOI:** 10.1097/PR9.0000000000000728

**Published:** 2019-04-16

**Authors:** Steven P. Cohen, Eric S. Schwenk, Eugene R. Viscusi

**Affiliations:** a Departments of Anesthesiology and Critical Care Medicine, Neurology and Physical Medicine & Rehabilitation, Johns Hopkins School of Medicine, Baltimore, MD; b Departments of Anesthesiology and Physical Medicine & Rehabilitation, Uniformed Services University of the Health Sciences, Bethesda, MD; c Department of Anesthesiology, Jefferson Medical College, Philadelphia, PA

We read with interest the recent article on ketamine by Bell and Kalso.^1^ In recent years, the use of ketamine to treat chronic pain and depression has exploded, and this article represents an easy-to-follow overview of this often misunderstood medication.

In their article, the authors focus on systematic reviews to draw broad conclusions on acute and chronic pain, without delving into the specifics of studies, including selection criteria and limitations. Although this methodology is sufficient for a topical review that summarizes basic information to include mainstream generalizations about efficacy and side effects, it is not capable of providing clinicians with practical recommendations that can be used to guide care such as indications and contraindications, dose regimens, benefit as an adjunct to opioids for acute pain, evidence for follow-up therapy with oral ketamine and other *N*-methyl-d-aspartate antagonists, how often and in whom to repeat procedures, how to prevent and treat adverse effects, and practical concerns such as how and by whom these patients should be monitored.

For both acute and chronic pain, this information and more is provided in recent guidelines along with a narrative review developed in a joint effort by the American Society of Regional Anesthesia & Pain Medicine, the American Academy of Pain Medicine, and the American Society of Anesthesiologists.^2,3^ Considering that anesthesiologists (not to mention other clinicians) comprise a plurality of International Association for the Study of Pain membership, these pragmatic concerns have clinical relevance for the Journal's readership. We assume the time frame of this publication, which was revised and accepted in June 2018, prevented the inclusion of these guidelines, but would encourage readers interested in the practical elements of intravenous ketamine use for pain treatment to explore these resources.

## Disclosures

The authors have no conflict of interest to declare.

The opinions or assertions contained herein are the private views of the authors and are not to be construed as official or as reflecting the views of the Department of the Army or the Department of Defense.
